# Exploiting Genomics Resources to Identify Candidate Genes Underlying Antioxidants Content in Tomato Fruit

**DOI:** 10.3389/fpls.2016.00397

**Published:** 2016-04-08

**Authors:** Roberta Calafiore, Valentino Ruggieri, Assunta Raiola, Maria M. Rigano, Adriana Sacco, Mohamed I. Hassan, Luigi Frusciante, Amalia Barone

**Affiliations:** ^1^Department of Agricultural Sciences, University of Naples Federico IIPortici, Italy; ^2^Department of Genetics, Faculty of Agriculture, Assiut UniversityAssiut, Egypt

**Keywords:** ascorbic acid, total carotenoids, *Solanum pennellii*, wild alleles, introgression sub-lines, *L-ascorbate oxidase*, *9-cis-epoxycarotenoid dioxygenase*

## Abstract

The tomato is a model species for fleshy fruit development and ripening, as well as for genomics studies of others Solanaceae. Many genetic and genomics resources, including databases for sequencing, transcriptomics and metabolomics data, have been developed and are today available. The purpose of the present work was to uncover new genes and/or alleles that determine ascorbic acid and carotenoids accumulation, by exploiting one *Solanum pennellii* introgression lines (IL7-3) harboring quantitative trait loci (QTL) that increase the content of these metabolites in the fruit. The higher ascorbic acid and carotenoids content in IL7-3 was confirmed at three fruit developmental stages. The tomato genome reference sequence and the recently released *S. pennellii* genome sequence were investigated to identify candidate genes (CGs) that might control ascorbic acid and carotenoids accumulation. First of all, a refinement of the wild region borders in the IL7-3 was achieved by analyzing CAPS markers designed in our laboratory. Afterward, six CGs associated to ascorbic acid and one with carotenoids metabolism were identified exploring the annotation and the Gene Ontology terms of genes included in the region. Variants between the sequence of the wild and the cultivated alleles of these genes were investigated for their functional relevance and their potential effects on the protein sequences were predicted. Transcriptional levels of CGs in the introgression region were extracted from RNA-Seq data available for the entire *S. pennellii* introgression lines collection and verified by Real-Time qPCR. Finally, seven IL7-3 sub-lines were genotyped using 28 species-specific markers and then were evaluated for metabolites content. These analyses evidenced a significant decrease in transcript abundance for one *9-cis-epoxycarotenoid dioxygenase* and one *L-ascorbate oxidase homolog*, whose role in the accumulation of carotenoids and ascorbic acid is discussed. Comprehensively, the reported results demonstrated that combining genetic and genomic resources in tomato, including bioinformatics tools, was a successful strategy to dissect one QTL for the increase of ascorbic acid and carotenoids in tomato fruit.

## Introduction

In recent years increasing attention has been given to the nutritional properties of plant antioxidant compounds, since their consumption has demonstrated to be associated with a reduced risk of cancer, inflammation and cardiovascular diseases. A great contribute to these health effects is attributed to secondary metabolites, including ascorbic acid (AsA, vitamin C) and carotenoids (precursors of Vitamin A) (Canene-Adams et al., 2005; Raiola et al., 2014). Besides their critical role in human nutrition, these compounds have major roles in several plant biological processes, such as photoreception and photoprotection, hormone signaling, cell cycle, cell expansion, plant development, responses to biotic and abiotic stresses. The biosynthetic pathway of carotenoids has been extensively studied and most metabolic key-steps that control their accumulation in plants has been identified (Giuliano, 2014). Plants produce AsA through several biosynthetic pathways, including the D-mannose–L-galactose as the main pathway, even though the role of the L-gulose, the D-galacturonate, and the *myo*-inositol pathways has also been suggested (Valpuesta and Botella, 2004); in addition the recycling pathway can contribute to the regulation of AsA accumulation (Chen et al., 2003). Finally, since AsA doesn’t diffuse through lipid bilayers because of its negatively charged form at physiological pH values, a class of transporters (Nucleobase Ascorbate Transporter, NAT) may be involved in the mechanisms of AsA accumulation (Badejo et al., 2012; Cai et al., 2014). The level of antioxidants in plants is highly influenced by environmental conditions, and this can explain why in recent years many scientific efforts were focused on better understanding the genetic architecture of this complex trait in various plant species (Davey et al., 2006; Stevens et al., 2007; Hayashi et al., 2012; Fantini et al., 2013; Kandianis et al., 2013; Lisko et al., 2014). Indeed, even though the biosynthesis of carotenoids and AsA in plants is well characterized, their gene regulation and their accumulation in fruits still remain elusive.

Humans are unable to synthetize AsA and carotenoids, and their dietary intake mainly derives from fruit and vegetables. Among these, tomato is the second most consumed vegetable in the world, thus being one of main sources of antioxidants. Indeed, tomato consumption reaches 40–45 kg *pro capita* per year in countries such as Spain, Italy, or USA (source: FAO databases); used as fresh product or processed (paste, juice, sauce and powder), its antioxidant content may protect against cancer, inflammation and cardiovascular diseases (Canene-Adams et al., 2005; Friedman, 2013).

Tomato is also a reference species for genetic and genomic studies in the Solanaceae family, due to its diploid genome with relative small size (950 Mbp), its short generation time, efficient transformation technologies, high synteny with various Solanaceae and numerous genetic and genomics resources already available (Mueller et al., 2005; Barone et al., 2008). Information data on gene function, genetic diversity and evolution in tomato and in other Solanaceae species are available since the year 2012 when the tomato genome was completely sequenced (Tomato Genome Consortium, 2012). Since then, high-throughput datasets and bioinformatics platforms extremely useful for the Solanaceae plant research community were newly generated or implemented. The Sol Genomics Network^1^ is a clade-oriented database for the Solanaceae family and its close relatives, which hosts genotypic and phenotypic data and analysis tools. The tomato genome resources database (TGRD^2^) is a resource that allows investigations on genes, quantitative trait loci (QTL), miRNA, transcription factors (TFs), single sequence repeat (SSR) and SNPs. Other specific databases, generated before the release of the tomato genome, are the SolEST, miSolRNA, Tomatoma, KaTomics, Tomato Functional Genomics Database (TFGD) and several others recently reviewed in Suresh et al. (2014).

Some of these resources might be extremely useful to dissect genetic complex traits into quantitative trait loci, especially when combined with the exploitation of genetic resources, such as the introgression lines (IL). These lines contain a defined homozygous segment of wild genome in a cultivated genetic background and, taken all together, represent a genomic library of the wild species (Eshed and Zamir, 1995). IL populations have been obtained from various wild tomato species, such as *Solanum pennellii, S. habrochaites, S. pimpinellifolium, S. lycopersicoides, S. chmielewskii*, and *S. sitiens* (Fernie et al., 2006) and they are useful to identify genes involved in QTLs regulation thus helping the detection of favorable wild alleles controlling the trait under study. The *S. pennellii* IL population is the most exhaustive; it consists of 76 lines with overlapping wild segments in the cultivated genetic background of the variety M82. These ILs have been widely used to map QTLs (Lippman et al., 2007), have been characterized at genomic and transcriptomic level (Chitwood et al., 2013) and, recently, Alseekh et al. (2013, 2015) carried out their high-dense genotyping and detailed metabolic profiling.

In this work we integrated genomic and transcriptomic data to identify candidate genes (CGs) controlling antioxidant metabolite accumulation in the fruit of *S. pennellii* IL7-3, which has been previously selected in our laboratory since it harbors a positive QTL for AsA and carotenoids content in the fruit (Sacco et al., 2013; Rigano et al., 2014). In addition, in order to restrict the number of CGs, we selected sub-lines of IL7-3 by the aid of species-specific CAPS markers and evaluated their metabolites content. This allowed us to identify one gene that might control carotenoids levels in the fruit. In addition, we could locate the genes controlling AsA content in a restricted part of the introgressed region 7-3, focusing on the role of one gene involved in AsA recycling pathway. These findings can provide valuable tools for improving the nutritional value of tomato and may represent a focus for future investigations.

## Materials and Methods

### Plant Material

Plant material consisted of one *S. pennellii* in *S. lycopersicum* introgression line (IL7-3, accession LA4102) and the cultivated genotype M82 (accession LA3475). The accessions were kindly provided by the Tomato Genetics Resources Centre^3^. Sub-lines of the region 7-3 (genotypes coded from R200 to R207) were selected from F_2_ genotypes previously obtained by intercrossing two ILs (IL12-4 × IL7-3; Sacco et al., 2013). The F_2_ genotypes were selfed for two generations and then screened by species-specific markers in order to select sub-lines carrying different wild regions at the homozygous condition. Additional IL7-3 sub-lines (genotypes coded from R176 to R182) were kindly provided by Dr. Dani Zamir (Hebrew University, Israel). All genotypes were grown in open-field conditions in the years 2014 and 2015 in a randomized complete block design with three replicates *per* genotype and 10 plants *per* replicate. Fruits were collected at three developmental stages (MG: mature green, BR: breaker stage, MR: mature red). Seeds and columella were subsequently removed, and fruits were ground in liquid nitrogen and stored at -80°C until analyses.

### Phenotypic Evaluations

#### Ascorbic Acid Determination

Ascorbic acid determination was carried out by a colorimetric method (Stevens et al., 2006) with modifications reported by Rigano et al. (2014). Briefly, 500 mg of frozen powder were extracted with 300 μl of ice cold 6% TCA. The mixture was vortexed, incubated for 15 min on ice and centrifuged at 14000 rpm for 20 min at 4°C. Twenty microliters of supernatant were placed in an eppendorf tube with 20 μl of 0.4 M phosphate buffer (pH 7.4) and 10 μl of double distilled (dd) H_2_O. Then, 80 μl of color reagent solution were prepared by mixing solution A [31% H_3_PO_4_, 4.6% (w/v) TCA and 0.6% (w/v) FeCl_3_] with solution B [4% 2,2′-dipyridil (w/v)]. The mixture was incubated at 37°C for 40 min and measured at 525 nm by a NanoPhotometer^TM^ (Implen). Three separated biological replicates for each sample and three technical assays for each biological repetition were measured. The concentration was expressed in nmol of AsA according to the standard curve, designed over a range of 0–70 nmol; then the values were converted into mg/100 g of fresh weight (FW).

#### Carotenoids Determination

The extraction of carotenoids was carried out according to the method reported by Zouari et al. (2014) with minor modifications. Briefly, one gram of frozen powder was extracted with a solution of acetone/hexane (40/60, v/v) for 15 min. The mixture was centrifuged at 4000 rpm for 10 min and the absorbance of surnatant was measured at 663, 645, 505, and 453 nm. Total carotenoids were determined by the equation reported by Wellburn (1994). Results were expressed as mg *per* 100 g FW. All biological replicates *per* sample were analyzed in triplicate.

### Molecular Marker Analysis

In order to define the wild region size of IL sub-lines, polymorphic markers spanning the introgression region 7-3 were searched for by exploring the Sol Genomics Network database^4^. Some markers were retrieved from the database, others markers instead were designed by searching for polymorphisms between the reference tomato sequence (release SL2.50) and the *S. pennellii* genome (Bolger et al., 2014) using the Tomato Genome Browser^5^. The primer pairs used to amplify the genomic region were designed using the Primer3web^6^. Total genomic DNA was extracted from leaves using the PureLink^TM^ Genomic DNA Kit (Invitrogen). PCR DNA amplification was carried out in 50 μl reaction volume containing 50 ng DNA, 1X reaction buffer, 0.2 mM each dNTP, 1.0 mM primer and 1.25 U GoTaq polymerase (Promega). Discriminating restriction enzymes were identified using the CAPS Designer tool available at the Sol Genomics Network^7^. The restriction endonuclease reaction was made in 50 μl of reaction volume containing 20 μl PCR product, 5 μl 10X reaction buffer and 1 μl of the selected restriction enzyme (10 u/ml). Digested fragments were separated by electrophoresis on 2% agarose gel in TAE buffer.

### Bioinformatic Identification of Candidate Genes

The search for CGs associated with ascorbic acid and carotenoids metabolism was conducted by exploring the annotations and the Gene Ontology terms of the genes included in the region 7-3 of the tomato chromosome 7 (Alseekh et al., 2013). Due to the preliminary annotation of *S. pennelliii* genome (Bolger et al., 2014), the genes of the wild parent were computationally re-annotated by Blast2Go program (version 3^8^; Conesa and Götz, 2008), to better characterize the gene set and collect additional information on their function. BlastX algorithm (e-value < 1E^-6^) and NCBI nr protein database were considered for Blast2Go analysis, while the annotation of all the sequences was performed by using default parameters (e-value < 1E^-5^). The ‘Augment Annotation by ANNEX’ function was also used to refine annotations (implemented in Blast2Go and described in Zdobnov and Apweiler, 2001).

Variants between *S. lycopersicum* and *S. pennellii* for all the CGs were obtained by extracting information from the Tomato Variant Browser (Aflitos et al., 2014). In addition, in order to validate the structural variants, the gene sequences were aligned using the genomic sequence information available for both cultivated *S. lycopersicum* and wild *S. pennellii* species. The effects of these variants and the prediction on their functional impact on the protein were analyzed using SnpEff v4.2 (Cingolani et al., 2012). The program performs a simple estimation of putative deleteriousness of the variants, classifying them in four classes (HIGH, MODERATE, LOW, MODIFIER, for detailed information refer to the documentation at http://snpeff.sourceforge.net/SnpEff_manual.html). Variants with high impact cause a stop codon or a frame shift; those with moderate impact are missense variants, whereas those with low impact are synonymous SNPs. The potential effect of these polymorphisms on the protein sequence was also cross-validated with the PROVEAN protein tool (publicly available from the J. Craig Venter Institute at http://provean.jcvi.org/seq_submit.php). According to the author’s guideline, we considered a “deleterious” effect of the variant if the PROVEAN score was equal or below -2.5.

The Tomato Functional Genomic Database (TED^9^), which reports RNA-seq data from the red fruit of *S. pennellii* ILs, was exploited to verify the expression of the identified CGs in tomato fruits and to estimate their differential expression in M82 and IL7-3. Finally, the TFs mapping in the introgression region were identified by investigating the 2505 TFs present in the Tomato Genomic Resources Database^10^ and the 1845 TFs categorized in the Plant Transcription Factor Database^11^. CGs and TFs with an RPKM value <3 in the RNA-seq database were excluded from further analyses and genes/TFs with Log_2_ ratio (IL7-3/M82) >1.5 or < -1.5 were considered to be differentially expressed, following the thresholds reported by Ye et al. (2015).

### Real-Time PCR Amplification of Candidate Genes

Total RNA was isolated from tomato fruit at the three stages of ripening (MG, BR, MR) by TRIzol^®^ reagent (Invitrogen, Carlsbad, CA, USA) and treated with RNase-free DNase (Invitrogen, Carlsbad, CA, USA; Madison, WI, USA) according to the method reported by the manufacturer (Invitrogen). Total RNA (1 μg) was treated by the Transcriptor High Fidelity cDNA Synthesis Kit (Roche) and cDNA was stored at -20°C until RT-PCR analysis. For each PCR reaction, 1 μL of cDNA diluited 1:10 was mixed with 12.5 μL SYBR Green PCR master mix (Applied Biosystems) and 5 pmol each of forward and reverse primers (Supplementary Table S1) in a final volume of 25 μL. The reaction was carried out by using the 7900HT Fast-Real Time PCR System (Applied Biosystems). The amplification program was carried out according to the following steps: 2 min at 50°C, 10 min at 95°C, 0.15 min at 95°C, and 60°C for 1 min for 40 cycles, and followed by a thermal denaturing step (0.15 min at 95°C, 0.15 min at 60°C, 0.15 min at 95°C) to generate the dissociation curves in order to verify the amplification specificity. All the reactions were run in triplicate for each of the three biological replicates and a housekeeping gene coding for the *elongation factor 1-α* (*Ef 1-α*) was used as reference gene. The level of expression relative to the reference gene has been calculated using the formula 2^-ΔCT^, where ΔCT = (CT_RNAtarget_ – CT_referenceRNA_) (Schmittgen et al., 2004). Comparison of RNA expression was based on a comparative CT method (ΔΔCT) and the relative expression has been quantified and expressed according to log_2_RQ, where RQ was calculated as 2^-ΔΔCT^, and ΔΔCT = (CT_RNAtarget_ – CT_referenceRNA_) – (CT_calibrator_ – CT_referenceRNA_) (Winer et al., 1999; Livak and Schmittgen, 2001). M82 MG, BR, and MR were selected as calibrators for the three analyzed stages of ripening. Quantitative results were expressed as the mean value ± SE. Differences among samples were determined by using Statistical Package for Social Sciences (SPSS) Package 6, version 15.0. Significance was determined by comparing the genotypes for each stage of ripening through a *t*-Student’s test at a significance level of 0.05.

## Results

### Phenotypic Evaluation of Parental Lines

**FIGURE 1:**
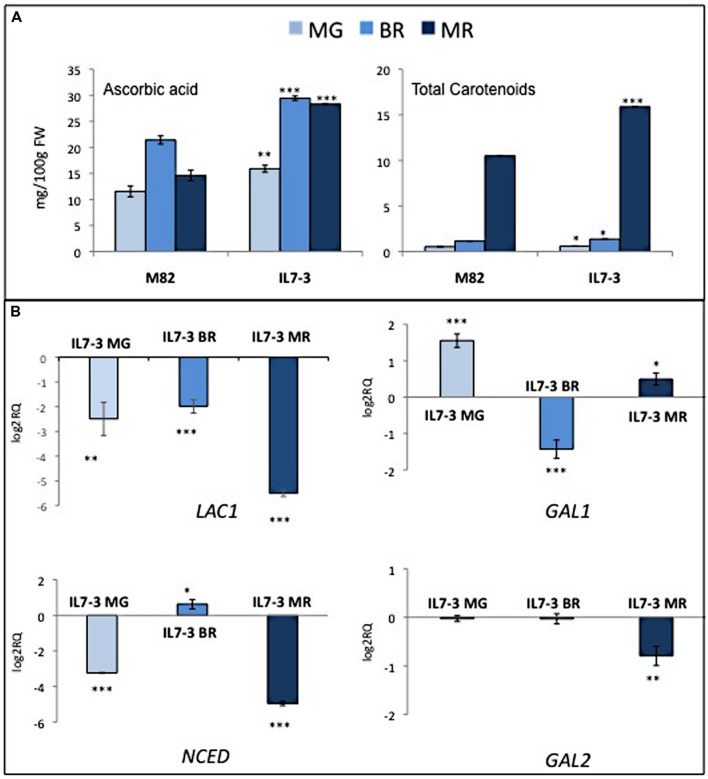
**Phenotypic and molecular evaluation of the parental genotypes M82 and IL7-3. (A)** Metabolite content (AsA and total carotenoids) at three different ripening stages (MG, mature green; BR, breaker; MR, mature red); **(B)** Expression level of four selected CGs (*LAC1*: *laccase-22/L-ascorbate-oxidase homolog*; *GAL1* and *GAL2*: *β-1-3-galactosiltrasferase*; *NCED*: *9-cis-epoxycarotenoid dioxygenase*) in IL7-3 at different ripening stages. Asterisks indicate statistically significant differences of each ripening stage to the corresponding M82 stage (^∗^*P* < 0.05, ^∗∗^*P* < 0.01, ^∗∗∗^*P* < 0.001).

In order to confirm the presence of one positive QTL for AsA and carotenoids in the region 7-3, metabolic analyses were performed for two consecutive years on mature red fruits of the cultivated genotype M82 and of IL7-3 grown in open fields (**Table 1**). In both years, IL7-3 accumulated a significant higher level of AsA and carotenoids in the fruit compared to M82, confirming data previously reported in our laboratory (Di Matteo et al., 2010; Sacco et al., 2013; Rigano et al., 2014). The metabolites content was also estimated in three different ripening stages (mature green – MG, breaker – BR, and mature red – MR) as shown in **Figure 1**. In M82, the AsA level increased from MG to BR and then decreased from BR to MR; accordingly, in IL7-3 the AsA level increased in the first ripening stages but did not decrease in MR. The total carotenoids content deeply increased in both genotypes from BR to MR as expected, and was higher in IL7-3.

**Table 1:** Evaluation of metabolite content (ascorbic acid, and total carotenoids, mean and standard error) in mature red fruit of genotypes M82 and IL7-3 in the years 2014 and 2015.

Genotype	Ascorbic acid		Total carotenoids	
	(mg/100 g FW)		(mg/100 g FW)	
	2014	2015	2014	2015
M82	14.62 ± 1.02	21.38 ± 2.13	10.62 ± 0.44	9.49 ± 0.61
IL7-3	28.32 ± 0.10^∗∗∗^	31.28 ± 1.71^∗∗∗^	15.88 ± 0.43 ^∗∗∗^	11.36 ± 0.62^∗∗^

*Asterisks indicate statistically significant differences compared to M82 (Student’s *t*-test, ^∗∗^*P* < 0.01, ^∗∗∗^*P* < 0.001)*.

### Identification of Candidate Genes (CGs)

In order to identify CGs controlling AsA and carotenoids content in IL7-3, we firstly better defined the introgression region size. At this purpose, we selected species-specific molecular markers at the two region borders, referring to those reported in the Sol Genomics Network database^12^ and taking into account the information on *S. pennellii* ILs reported in Chitwood et al. (2013) regarding the chromosomal positions of ILs boundaries. By testing ten markers (from N22 to N28 at the upper border, and from N12 to N30 at the lower border, **Table 2**) on the parental genotypes M82 and on IL7-3, we ascertained that the wild region stretches from marker N27 (corresponding to Solyc07g048030 at 59,218,716 bp) to marker N17 (corresponding to Solyc07g063330 at 65,816,155 bp), covering about 6.6 Mbp. This region includes 725 genes (Supplementary Table S2), 120 (16.5%) were annotated as unknown proteins, whereas 94 (13.0%) were TFs. Among the remaining 511 annotated genes, we searched for those related to AsA and carotenoids accumulation. Six CGs putatively involved in determining AsA content were detected (**Table 3**), but none of them belong to the main biosynthetic galactose pathway. The identified genes were: one *polygalacturonase* (Solyc07g056290, *POLYGAL*), two *beta-1-3-galactosyltransferase* (Solyc07g052320 and Solyc07g062590, *GAL1* and *GAL2*, respectively), two *laccase-22/L-ascorbate-oxidase homolog* (Solyc07g052230 and Solyc07g052240, *LAC1* and *LAC2*, respectively), and one *nucleobase-ascorbate transporter* (Solyc07g049320, *NAT*). The investigation of the SolCyc biochemical pathways database^13^ allowed confirming the involvement of the gene *POLYGAL* in the galacturonate AsA biosynthetic pathway (enzymatic step EC 3.2.1.15), and of *GAL1* and *GAL2* in enzymatic reactions (EC 2.1.4-) potentially regulating *myo*-inositol content, that might feed the glucuronate biosynthetic pathway. *LAC1* and *LAC2* might enter the recycling pathway of AsA by reducing L-ascorbate into monodehydroascorbate (EC 1.10.3.3), whereas the NAT might have a role in transporting AsA among the different intracellular compartments. In addition, in the introgression region one *9-cis-epoxycarotenoid dioxygenase* (Solyc07g056570, *NCED*) was also mapped that, entering the carotenoids pathway, determines the carotenoids oxidative cleavage with consequent production of apocarotenoids, the direct substrates for abscisic acid (ABA) synthesis. The latter gene was included into the group of those to be further investigated.

**Table 2:** CAPS molecular markers used to define the whole introgression region 7-3 and the sub-lines obtained from this region.

Marker code	Position on Chromosome 7 SL2.5 (bp)	Primer sequence 5′-3′	Expected PCR product size (bp)	Restriction enzyme	M82 fragment size (bp)	IL7-3 fragment size (bp)
N22	58,951,964	F:ATGTGCTTGCCATGTGTCTG	507	*Taq*I	290 + 217	507
		R:AAGAGATGGAGCGTTTGGGA				
N23	58,963,247	F:TGACCACTGCCCTAATGCTT	526	*Hae*III	288 + 238	526
		R:GCTGATGAAGTGAGGAACCC				
N24	59,131,334	F:CACAGTCATCTTCAGCAATGTG	444/481	*Rsa*I	90 + 391	90 + 348 + 43
		R:CTTGTCTTCCCATAGCTGCG				
N26	59,184,600	F:GATGGTAGTTTCATGCGGATCA	378	*Taq*I	296 + 47 + 35	343 + 35
		R:GTCCACCTGCTAACCTCAGT				
**N27**	59,218,716	F:TGGGACACAAATGAAGAGCG	610	*Taq*I	610	291 + 319
		R:ACTGTGGATGCTAAACCTCCA				
N28	59,240,752	F:CAGCAATAACCAGATTTTCGCA	402	*Hae*III	402	268 + 134
		R:CCAGCAACAACAGCACCATT				
N25	59,289,774	F:TGTCACTGGTTCCTTCATCAAC	612	*Taq*I	417 + 195	612
		R:GCGGAAAGGCAAACTCCAAA				
N14	59,523,504	F:TCCGCTCTTCATCATCTGTTG	492	*Taq*I	492	399 + 93
		R:TCCAATTCCATCCCGATTTGC				
N18	59,578,421	F:GCCATTTAACATTGGGACTCG	440	*Sca*I	223 + 217	440
		R:AGCTTACATCTGATCCGCCC				
N15	59,926,751	F:TGACATGCCGATAGTGTTCAC	489	*Rsa*I	489	176 + 313
		R:TGTGATGGTGTTTGACTGGG				
N16	60,507,445	F:CGCTTGCCCTTTGTAATCCA	869	*Rsa*I	57 + 483 + 173 + 156	57 + 483 + 329


		R:ACTGGTGGGACGTATACTTTGT				
N33	60,724,902	F:ACAGTGTGAGTCCCTTCTACT	650	*Alu*I	650	257 + 393
		R:AATTGTCCCATTCCACCAGG				
N10	60,874,313	F:GATTGCTGGTCTACGCTTGC	303	*Taq*I	263 + 40	56 + 96 + 111 + 40
		R:ACAAGAAGCCAGCAAAGACG				
N11	61,065,289	F:GCTTCCTCAAGACACCCAGA	458	*Hae*III	458	304 + 154
		R:CAGTTGTTCATTCAGTCAGGCT				
N4	61,181,115	F:CAATGAGATATACTGGGTACACG	782	*Taq*I	414 + 176 + 192	590 + 192


		R:AACGTGCAGAGAACAAAGTTGAG				
N1	62,747,850	F:TGACGCGATAAACCTTGAGCAGCAC	300	*Taq*I	300	300 + 100
		R:ATAACCTAGCTCCCTCCTTATGGTGTC				
N8	63,198,615	F:GGTGGCAATTAAGGGTGACA	767	*Hae*III	518 + 251	767
		R:TCAAAATCCACCGTACACCA				
N19	64,147,505	F:GGATGGACAAGGTGCTGTTG	824	*Sca*I/*Rsa*I	139 + 685	824
		R:TTCTGTTCATATCCGTCGTTCA				
N9	64,214,216	F:GCACGAAACCCCACCAATTA	743/–	–	743	–
		R:GCAATCTCCAGTAGTATGTGAG				
N2	64,340,348	F:TCACTTCTTGTATTTGTGCAGAGG	650	*Taq*I	420 + 150 + 80	500 + 150
		R:AGTGCCCTTATGTTAAGCTTTATCC				
N5	64,734,536	F:TAGAGGACGGGGAATGGACC	854	*Alu*I	854	586 + 268
		R:AAGGAGGGAAGAGGCTTGTG				
N6	65,022,435	F:GTCGAACCTTGATTTTACCTGG	967/947	–	967	947
		R:GACTGACATATGCTCTGCTTCA				
N7	65,309,240	F:AACAGGGTGGTGGTAGATGG	609	*Taq*I	609	422 + 187
		R:TCAACCATGCGTGTTATTAGCA				
N3	65,598,163	F:TTGGTCTATTTGCAATATTTGATGG	370	*Alu*I	370	230 + 140
		R:AATCAATATGGCTGTAACAGCAGTTG				
N12	65,699,488	F:GGGATCGTTGTTGCTGGTTC	301	*Hae*III	301	168 + 133
		R:GCCATTGTCTCACCGAGCT				
**N17**	65,816,155	F:CCAATCCTAGTATACCTCCAGCA	413	*Eco*RI	413	241 + 172
		R:TGAATATGCCATGCGAAGTTGT				
N29	65,969,635	F:AGATGAGCAGTTGGGTAGTCC	450	*Taq*I	270 + 180	450
		R:CCAAAAGCCATCAGTTGCCT				
N30	66,061,621	F:AGTAGAACCAGAGGATAGGGAAC	520	*Taq*I	297 + 223	520
		R:GGAGTAGAGGCAGCAATGGA				

*Markers are ordered following their crescent map position (bp) on chromosome 7. For each marker are reported: the primer sequences, the PCR product size, the restriction enzymes used, the polymorphic fragment size between M82 and IL7-3. In bold: markers that are borders of the introgression region*.

**Table 3:** Candidate genes for ascorbic acid and carotenoids, and transcription factors mapping in the introgressed region 7-3, all selected for their log_2_ fold change <-1.5 or >1.5.

Gene function	Gene identifier (Solyc ID)	Gene position (SL2.50) bp	Expression level RPKM^1^		Log_2_ fold change	Prediction by SNPeff			Prediction by PROVEAN
			M82	IL7-3		Variants with HIGH impact No.	Variants with MODERATE impact No.	Variants with LOW impact No.	Deleterious variants No.
**Candidate genes**								
*Nucleobase ascorbate transporter* (NAT)	Solyc07g049320	59577405…59581995	0.04	0.02	-1	0	0	10	–
*Laccase-22/L-ascorbate oxidase homolog* (LAC1)	Solyc07g052230	60723150…60726170	9.43	0.02	-8.8811	0	6	13	1
*Laccase-22/L-ascorbate oxidase homolog* (LAC2)	Solyc07g052240	60726910….60730555	0.04	0.00	ND	0	2	25	0
*β-1-3-galactosyltransferase* (GAL1)	Solyc07g052320	60816826…60820674	9.43	3.48	-1.4382	0	0	11	–
*Polygalacturonase* (POLYGAL)	Solyc07g056290	64146906…64150487	0.00	0.00	ND	0	7	9	1
*9-cis-epoxycarotenoid dioxygenase* (NCED)	Solyc07g056570	64361346…64363163	97.58	19.77	-2.3033	0	4	19	0
*β-1-3-galactosyltransferase* (GAL2)	Solyc07g062590	65282118…65288822	7.35	2.54	-1.5329	0	4	7	0
**Transcription factors**								
Storekeeper protein	Solyc07g052870	61299858…61301426	1.54	4.45	1.5308	0	2	1	0
GRAS family transcription factor	Solyc07g052960	61367195…61368484	451.9	1421.77	1.6533	0	3	7	0
CONSTANS-like zinc finger protein	Solyc07g053140	61593135…61594431	35.64	6.53	-2.4483	0	6	5	0
Myb-related transcription factor	Solyc07g053240	61710088…61711189	0.08	4.08	5.6724	1	8	4	0
Ethylene-responsive transcription factor 4	Solyc07g053740	62167919…62168596	222.36	15.85	-3.8103	0	0	11	–
Ethylene responsive transcription factor 2a	Solyc07g054220	62574142…62575314	1.37	0.43	-1.6717	0	10	6	0
WRKY transcription factor 5	Solyc07g055280	63369004…63372627	5.26	0.56	-3.2315	0	2	4	0
BHLH transcription factor	Solyc07g062950	65585877…65588912	2.55	0.62	-2.0401	0	1	3	0
Squamosa promoter binding-like protein	Solyc07g062980	65599440…65600609	55.86	2.15	-4.6994	0	2	2	0
DNA-binding WRKY VQ	Solyc07g063070	65649614…65650315	10.77	3.45	-1.6423	0	2	5	0

*For each Solyc the position on the chromosome is reported in bp. The number of sequence variants of wild alleles classified for their impact/deleteriousness following SNEeff and PROVEAN are reported. ^1^RPKM expression values are those reported in the Tomato Functional Genomic Database (http://ted.bti.cornell.edu)*.

### Sequence Variation and Expression Variability of Selected CGs

In order to better define which CGs determine the different metabolites content between M82 and IL7-3 fruit, differences in their sequence and/or in their expression level were investigated. The impact of polymorphisms between *S. lycopersicum* and *S. pennellii* was estimated for the 117 variations identified in CGs mapping in the introgression region (**Table 3**). No case of high impact polymorphism was detected, and *NAT* and *GAL1* did not even exhibit any variants with moderate impact effect. For the other genes, the number of variants with moderate impact varied from two (*LAC2*) to seven (*POLYGAL*), and a deleterious effect at the protein level investigated by PROVEAN was predicted for genes *LAC1* and *POLYGAL*.

The RNA-seq data available for M82 and IL7-3 in the TFGD (Fei et al., 2010) allowed to ascertain that three CGs for AsA were not expressed or expressed at very low levels in the red fruit (**Table 3**): *NAT*, *LAC2* and *POLYGAL*. By contrast, *GAL1*, *GAL2*, *LAC1* and *NCED* showed a lower expression level in IL7-3 compared to M82. The expression of all CGs was analyzed by qRT-PCR in three developmental stages of M82 and IL7-3 fruits. This analysis allowed confirming the lack of expression of *NAT*, *LAC2* and *POLYGAL*. The different expression levels of *LAC1* and/or *GAL2* detected here well correlate with the different trend of AsA accumulation in M82 and IL7-3 in the three developmental fruit stages (**Figure 1**), even though also the down-regulation of *GAL1* at BR stage could be relevant. Finally, the significant lower expression of the gene *NCED* at the MR stage well correlated with the higher level of total carotenoids observed in IL7-3.

Besides the identified CGs, TFs mapping in the introgression region 7-3 might play a role in increasing antioxidants. Indeed, if differentially expressed or polymorphic between M82 and IL7-3, they could *trans*-regulate the expression of genes involved in AsA or carotenoids biosynthesis and accumulation mapping in the introgression or in other regions of the genome. Sequence variations with deleterious effects on the protein functionality were found in 27 TFs (Supplementary Table S3), but in most cases (85%) the polymorphic TFs were not expressed in the fruit (Supplementary Table S4). The ten TFs selected for their significant differential expression between IL7-3 and M82 (Supplementary Table S3) did not show any sequence variation that cause deleterious effect as predicted by PROVEAN. Most of them exhibited a lower expression in IL7-3, except for one MYB (Solyc07g053240), one GRAS (Solyc07g052960) and one storekeeper protein (Solyc07g052870), but none corresponded to the TFs identified by Ye et al. (2015) for their correlation with expression of genes involved in high AsA and carotenoids in tomato fruit. The availability of the whole transcriptome of M82 and IL7-3 in the TED database allowed also investigating the expression of all the genes involved in AsA and carotenoids biosynthetic pathways, which were annotated in the tomato genome (Supplementary Table S5). No differentially expressed gene among these was identified. When looking at the whole transcriptome, an unbalance of ascorbate-oxidase activity could be hypothesized in IL7-3 compared to M82. Indeed, besides the gene *LAC1* of the introgression 7-3, two other *laccases-22/L-ascorbate-oxidase*, mapping on chromosomes 2 and 8, showed a decreased expression in IL7-3. They could exert an additive action to that of the wild *LAC1* in increasing AsA content in the fruit. Finally, two *myo-inositol phosphate synthase* (Solyc04g050820 and Solyc05g051850) were over-expressed in IL7-3, and they could affect the *myo*-inositol pathway, as well as the two *GAL1* and *GAL2* that map into the introgression.

### Selection and Phenotyping of IL7-3 Sub-lines

**FIGURE 2:**
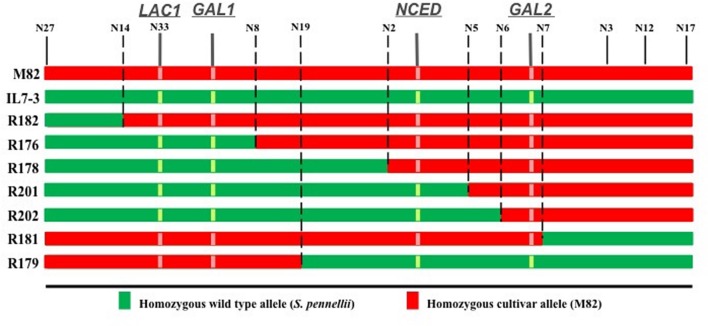
**Size and genomic identity of the seven sub-lines selected from the region 7-3**. For each sub-line (coded R), the boundary edges are indicated by the CAPS markers (coded N) that separated the wild genomic region (in green) from the cultivated one (in red). The position of the four CGs (*LAC1*, *GAL1*, *NCED*, *GAL2*) in the region 7-3 is also reported.

**FIGURE 3:**
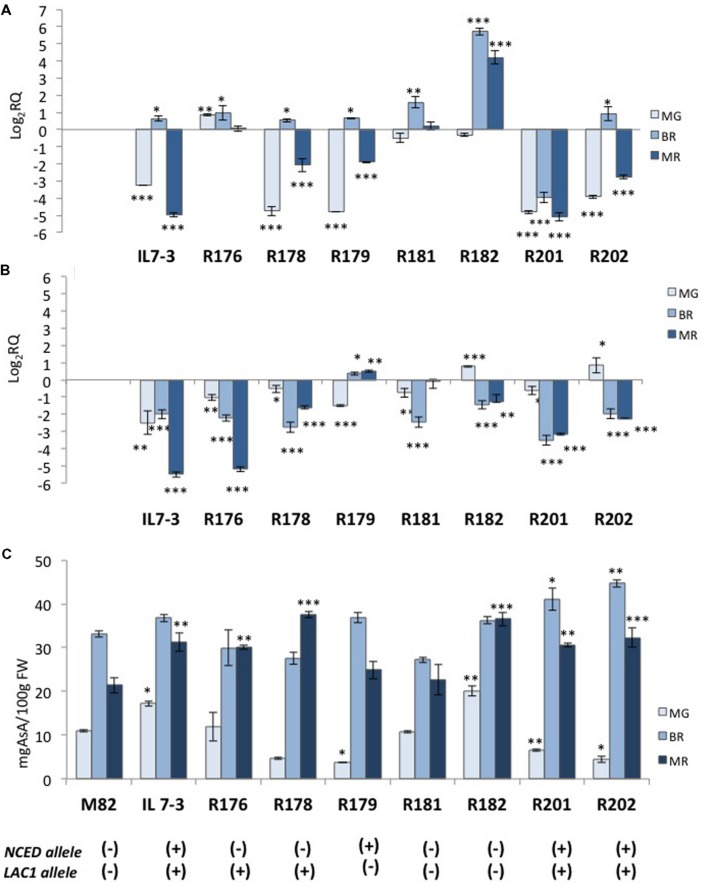
**Gene expression of two CGs (*NCED* and *LAC1*) and ascorbic acid (AsA) content in the fruit of the seven sub-lines of the region 7-3**. The expression level of *NCED* **(A)** and *LAC1* **(B)** in the parental IL7-3 and its sub-lines is reported in comparison to that observed in the parental M82 at three different ripening stages, together with the AsA content **(C)** in the fruit evaluated at the same stages for all genotypes. Asterisks indicate statistically significant differences of each ripening stage to the corresponding M82 stage (^∗^*P* < 0.05, ^∗∗^*P* < 0.01, ^∗∗∗^*P* < 0.001). The presence of the wild (+) or cultivated (-) alleles of *NCED* and *LAC1* genes for each genotype is reported at the bottom.

In order to reduce the number of CGs potentially responsible for the higher antioxidant content in the IL7-3 red ripe fruit, introgression sub-lines of the wild region were selected through the analysis of 28 polymorphic markers mapping within this region. Comprehensively, we identified seven distinct sub-lines showing a reduced wild region compared to IL7-3 and carrying different combinations of wild alleles for four CGs (**Figure 2**). Only two sub-lines, R182 (from marker N27 to N14) and R181 (from marker N7 to N17), had no wild alleles for the CGs, even though they carried different introgressed wild regions. Four sub-lines carried two wild CGs for AsA (*LAC1* and *GAL1*) but differed for the presence (R201, R202) or absence (R176, R178) of the wild allele for *NCED* gene. Finally, one sub-line (R179) carried wild alleles for *GAL2* and *NCED* genes. All the sub-lines were grown in open field and were evaluated for AsA and carotenoids content in the fruit (**Table 4**). The carotenoids level of the three sub-lines carrying the wild allele for *NCED* (R179, R201, R202) was higher than in M82; sub-lines carrying the cultivated allele showed a level of carotenoids comparable to M82. As for AsA, it was evident that two sub-lines (R179 and R181) exhibited levels of AsA comparable to that of the control genotype M82, whereas all the others showed AsA content significantly higher than M82 and similar to IL7-3. Therefore, considering the different combinations of wild CGs carried by the sub-lines, it is possible to exclude the role of *GAL2* wild allele in increasing AsA content in IL7-3. By contrast, the wild alleles of both *LAC1* and *GAL1* might be involved in increasing AsA in IL7-3 and in a group of sub-lines, even though the qRT-PCR results evidenced that the action of *GAL1* occurs earlier at BR. The expression level of the selected CGs *NCED* and *LAC1* was verified in the sub-lines (**Figure 3**) to better ascertain their role in affecting carotenoids and AsA, respectively. Surprisingly, a higher expression of *NCED* compared to M82 and a concurrent higher level of AsA were observed in R182, a sub-line with a reduced introgression size of 200 kb, where no wild allele for any CGs and no differentially expressed or polymorphic TFs was retained. A total of 24 genes map in this region, including two unknown genes.

**Table 4:** Phenotyping and genotyping of the seven *S. pennellii* introgression sub-lines: for each sub-line the higher content of ascorbic acid (AsA) and total carotenoids respect to M82 is reported, together with the presence of wild alleles for four candidate genes (CG), and the number of genes classified as unknown and transcription factors (TF), which map in the introgressed region.

Sub-line	Border Markers	AsA^1^	Carotenoids^1^	Wild CGs	Unknown	TFs
R182	N27-N14	+	-	–	2	0
R176	N27-N8	+	-	*LAC1*- *GAL1*	65	6
R178	N27-N2	+	-	*LAC1*- *GAL1*	85	7
R201	N27-N5	+	+	*LAC1*- *GAL1*-*NCED*	91	7
R202	N27-N6	+	+	*LAC1*- *GAL1*-*NCED*	100	7
R179	N19-N17	-	+	*GAL2*-*NCED*	36	3
R181	N7-N17	-	-	–	12	3

*^1^Ascorbic acid (AsA) and carotenoids content in the sub-line significantly higher (+) than M82*.

## Discussion

The exploitation of wild *Solanum* species has driven the improvement of tomato varieties for several traits by using traditional and innovative breeding approaches (Bai and Lindhout, 2007). The wild species are precious sources of new alleles for improving specific traits, most of which are quantitatively inherited and therefore highly influenced by environmental conditions and by multiple interactions among a consistent number of genes. In this view, the production of ILs from tomato wild species helped to dissect many complex traits into major QTLs (Lippman et al., 2007), which might be then transferred into improved varieties. This genetic effort is boosted by the availability of genomic tools and resources, which may have a deep impact on the success of this breeding strategy.

The *S. pennellii* IL7-3 has been selected in our laboratory for its higher antioxidant properties compared to the cultivated variety M82 (Sacco et al., 2013; Rigano et al., 2014). It exhibited stable performances in different years and we could therefore assume that these properties depend on a strong genetic basis. By integrating data coming from many genomics resources publicly available, we exploited this genetic resource with the aim of identifying CGs and their wild alleles, which may contribute to increase AsA and total carotenoids in the fruit. One CG was identified that might affect carotenoids accumulation: the *NCED* gene, which controls a key-enzyme in ABA biosynthesis (Zhang et al., 2009). The lower expression of this gene in IL7-3 might reduce the metabolic flux toward ABA production, pushing the upstream metabolic pathway and thus feeding carotenoids accumulation, as proposed by Sun et al. (2012), who observed an increase of carotenoids level and a reduction of ABA when the gene *SlNCED1* was silenced in tomato fruit. Also in our case, a concurrent increase of carotenoids and decrease of ABA was observed in IL7-3 compared to M82 (data not shown), thus supporting the role attributed to the wild allele of *NCED*. In addition, even though no deleterious impact on proteins was detected by PROVEAN when comparing the wild and cultivated alleles of *NCED*, the alteration of amino acid sequences may result in enzymes with modified activities (Yuan et al., 2015).

Understanding the genetic control of the higher AsA content in IL7-3 fruit was more complicated. Out of six CGs identified in the introgressed region, which might be involved in the synthesis and accumulation of AsA, only three were expressed in the fruit. Among these, the gene *LAC1* was expressed only in traces in IL7-3 fruits respect to M82, as retrieved from available RNAseq data in the TED database, and confirmed in our work using three primer pairs for Real Time PCR targeting different regions of this gene. In addition, a deleterious missense variant (-6.337 PROVEAN score) has been detected in *LAC1* when comparing the sequences of *S. lycopersicum* and *S. pennellii*. This caused a substitution of a glycine in glutamic acid at position 194 (G194E), which affects the cupredoxin domain. The deleterious alteration of the protein in that position might be crucial for its correct functionality. The two *GAL* genes mapping in the introgression exhibited both a slight lower expression in IL7-3, and might contribute to enhance AsA content in the fruit. Finally, the wild allele of *NCED* might be indirectly related to the increased level of AsA, since an intricated relationship between ABA biosynthesis and AsA metabolism has been already hypothesized in different species, including Arabidopsis, strawberry, tomato, and *Ocimum* (Ghassemian et al., 2008; Nair et al., 2009; Lima-Silva et al., 2012; Dongdong et al., 2015). However, in some cases the correlation occurred between ABA content and ascorbate oxidase expression level without any modifications of NCED expression (Lopez-Carbonell et al., 2006; Fotopoulos et al., 2008). Therefore, the AsA increase in IL7-3 might be also attributed to variations of ABA content determined by the reduced activity of NCED, LAC1 or both the enzymes.

Since the introgressed region 7-3 has a large size (6.6 Mbp), we analyzed sub-lines of the region, which allowed focusing on a restricted number of CGs. The selected sub-lines carried only two or three CGs, or even no CG at all. The phenotypic characterization of the sub-lines led us to draw some conclusions on the potential role of the CGs. Firstly, the carotenoids content well-reflected the presence/absence of the wild allele for *NCED*, even though its specific action should be further investigated, since the lower expression of *NCED* is correlated to a higher carotenoids content only in three out of four sub-lines (R179, R201, and R202). Therefore, approaches of gene replacement between the wild and the cultivated allele will be undertaken to verify that the presence of the wild allele may be effectively correlated to higher levels of carotenoids content. Secondly, the wild allele of *NCED* is not essential for determining the higher AsA content in IL7-3, even though it can contribute to increase it, as discussed above. Thirdly, the down-regulation of *GAL2* is not correlated to the higher AsA content in IL7-3 and in sub-lines.

Unfortunately, it was not possible to disrupt the linkage between the two CGs *LAC1* and *GAL1*, and then clearly identify if only one or both of them control the AsA increase. However, it is expected that more genes mapping in one QTL contribute to affect one phenotype, and therefore in our case it may be assumed that both *LAC1* and *GAL1* might have a determinant role in increasing AsA, and this could confirm the existence of polygenes in the QTL under study. In particular, the down-regulation of *GAL1* at BR stage would reduce the metabolic flux toward the *myo*-inositol biosynthetic pathway. Moreover, it is worth saying that, when the whole transcriptome of IL7-3 was analyzed in comparison to that of M82, two genes annotated as *inositol-3-phosphate synthase* were over-expressed in IL7-3, with a potential contrasting action on the *myo*-inositol pathway respect to *GAL1*. Since the involvement of the latter pathway in the AsA biosynthesis in plants is still controversial (Endres and Tenhaken, 2009; Torabinejad et al., 2009; Gallie, 2013), we did not focus further attention on *GAL1*.

The role of *LAC1* is supported by its down-regulation in IL7-3 MR fruit and in the sub-lines that exhibited a high level of AsA (R176, R178, R201, R202). Indeed, the ascorbate oxidase is an apoplastic enzyme that catalyzes the reversible oxidation of ascorbate to dehydroascorbate, through the formation of monodehydroascorbate, with the concomitant reduction of molecular oxygen to water. Transgenic plants over-expressing or under-expressing this gene have shed light on its role in regulating the apoplastic ascorbate pool (Pignocchi et al., 2003; Sanmartin et al., 2003), and therefore the ascorbate redox state, thus also influencing the perception of environmental stresses (Yamamoto et al., 2005; Fotopoulos et al., 2006, 2008; Garchery et al., 2013). In our case, the down-regulation of the wild *laccase-22/L-ascorbate oxidase LAC1* might have an effect comparable to that described in RNAi lines with reduced ascorbate oxidase activity (Zhang et al., 2011), which exhibited high ascorbic acid accumulation in tomato fruit. This effect could explain the significantly higher Asa level observed in IL7-3 and in the sub-lines with respect to the control M82. In the future, the potential correlation between the down-regulation of *LAC1* in our tomato genotypes and their improved response to agents imposing oxidative stress will be also investigated.

Finally, in the sub-line R182 the increased level of AsA detected in the red ripe fruits should be further investigated, but it confirms that the IL7-3 AsA QTL consists of more than one CG. Among the 24 genes mapping in the small introgressed region of R182, two annotated as unknown were detected, whereas no TFs was differentially expressed in tomato fruit. In the future, the functional role of all these 24 genes in modulating AsA in tomato fruit will be further investigated by using reverse genetic approaches to clearly define their specific role in controlling AsA content in the tomato mature fruit.

It is also worth saying that the existence in the 7-3 region of several genes annotated as unknown proteins paves the way to other hypotheses. Indeed, besides the differential expression and the structural variants affecting enzyme activity, additional transcriptional and translational interations may occur and contribute to influence it. Recently, regulators of AsA biosynthesis in plants have been described (reviewed in Zhang, 2012). In *Arabidopsis* it has been demonstrated that light regulates AsA synthesis through the interaction between a photomorphogenetic factor and the enzyme GDP-mannose pyrophosphorylase (Wang et al., 2013), as well as that a feedback regulation of Asa biosynthesis occurs following the interaction between ascorbate and an Open Reading Fame (ORF) in the long 5′ UTR (untranslated region) of the GDP-L-galactose phosphorylase gene (Laing et al., 2015). Now, it will be crucial to demonstrate if similar mechanisms or others may operate in tomato fruit and, therefore, influence AsA concentration in IL7-3.

## Conclusion

Results reported in the present work clearly demonstrated that exploiting the genetic and genomic resources nowadays available for tomato, including bioinformatics tools, was a successful strategy to dissect one positive QTL for the increase of AsA and carotenoids in the mature fruit. In particular, two CGs for improving these metabolites were detected in the wild region 7-3 introgressed from the species *S. pennellii*. These were one *L-ascorbate oxidase* (*LAC1*) and one *9-cis-epoxycarotenoid dioxygenase* (*NCED*), whose wild alleles, exhibiting polymorphisms and/or differential transcript levels, might increase AsA and total carotenoids content. The first CG favors the accumulation of reduced ascorbate controlling the redox state of ascorbate in the apoplast. The action of the second CG still needs to be elucidated, even though the presence of the wild allele for *NCED* was correlated to higher carotenoids content. Finally, the latter gene might also indirectly contribute to increase AsA content, as revealed by the sub-line R182, which showed a high expression of the cultivated allele for *NCED* combined with high AsA content. A group of 24 genes mapping in the wild introgression of the sub-line R182 will be further investigated in the future to better understand their role in the architecture of the QTL that positively influences the level of antioxidants in the investigated region of the chromosome 7.

## Author Contributions

RC and VR contributed to bioinformatic and experimental analyses carried out to identify CGs and the introgression sub-lines, and to draft the manuscript; AR contributed to metabolic and transcriptomic analyses; MR contributed to metabolic analysis and critically revised the manuscript; AS contributed to the bioinformatic analysis and drafted the manuscript; MH contributed to molecular marker analysis and to grow materials; LF contributed to the conception of the experiment and critically revised the manuscript; AB contributed to the experiment design, to data analysis and interpretation, to draft the manuscript.

## Conflict of Interest Statement

The authors declare that the research was conducted in the absence of any commercial or financial relationships that could be construed as a potential conflict of interest.

## Footnotes

1 https://solgenomics.net/

2 http://59.163.192.91/tomato2/

3 http://tgrc.ucdavis.edu/

4 https://solgenomics.net/

5 http://www.tomatogenome.net/VariantBrowser

6 http://primer3.ut.ee/

7 http://solgenomics.net/tools/caps_designer/caps_input.pl

8 https://www.blast2go.com/

9 http://ted.bti.cornell.edu/

10 59.163.192.91/tomato2/tfs.html

11 planttfdb.cbi.pku.edu.cn

12 solgenomics.net

13 solcyc.solgenomics.net
