# Supplementary material for: Exploiting Genomics Resources to Identify Candidate Genes Underlying Antioxidants Content in Tomato Fruit
Source: Front Plant Sci. 2016 Apr 8;7:397. doi: 10.3389/fpls.2016.00397 (PMC4824784; doi:10.3389/fpls.2016.00397)
Supplement: Supplementary file 1 [file Table_1.DOC]

**Supplementary Table 1** - Sequences of adopted primers for amplifying seven candidate genes and the housekeeping gene *EF-1α* by Real-Time qPCR. For the candidate gene *LAC1* three primer pairs were designed, targeting different regions: primers pairs coded LAC1-A, LAC1-B, LAC1-C

| **Gene** | **Primer** | **Sequence** |
| --- | --- | --- |
| *EF-1α* | Solyc06g005060FW | 5’-AAGCTGCTGAGATGAACAAG-3’ |
|  | Solyc06g005060RV | 5’-GTCAAACCAGTAGGGCCAAA-3’ |
| *NAT* | Solyc07g049320FW | 5’-AGCTCGTCATGGTCTTGTTC-3’ |
|  | Solyc07g049320REV | 5’-TGGAGGCGATGTGAATATGG-3’ |
| *POLYGAL* | Solyc07g056290FW | 5’-AGATGAAAGCCCTAACACGG-3’ |
|  | Solyc07g056290REV | 5’-AGGTCCACACGTAACATTGG-3’ |
| *NCED* | Solyc07g056570FW | 5’-CGACAAGAACAAAGTTTCCCG-3’ |
|  | Solyc07g056570REV | 5’-TTCCCAAGCATTCCAGAGG-3’ |
| *GAL1* | Solyc07g52320FW | 5’-TCAGTTCGTGCTACATGGATG-3’ |
|  | Solyc07g52320REV | 5’-TGATTTCTGTCCTCGGCTTC-3’ |
| *GAL2* | Solyc07g62590FW | 5’-TCCTCAACTATCTACTCCCGC-3’ |
|  | Solyc07g62590REV | 5’-TTGACCCTAAACCCTGCTTC-3’ |
| *LAC1-*A | Solyc07g052230FW | 5’-GTAGTTGAAGTAGAAGGGACTCAC-3’ |
|  | Solyc07g052230REV | 5’-ACCTGAGATGTGAAACGAGTG-3’ |
| *LAC1-*B | Solyc07g052230FW | 5’-CATGGCAGGATGGAGTCTATG-3’ |
|  | Solyc07g052230REV | 5’-GCAAGGGAAGGAAAGTAGTAGAA-3’ |
| *LAC1-*C | Solyc07g052230FW | 5’-GGCGTAATCTAACAGCTA-3’ |
|  | Solyc07g052230REV | 5’-CAAGTCTAATTGTGCGTG-3’ |
| *LAC2* | Solyc07g052240FW | 5’-GACAATGTGGGAATGTGGAAC-3’ |
|  | Solyc07g052240REV | 5’-GACAGGTGAATAAACGCGAAG-3’ |
